# Synergistic Nanomedicine: Photodynamic, Photothermal and Photoimmune Therapy in Hepatocellular Carcinoma: Fulfilling the Myth of Prometheus?

**DOI:** 10.3390/ijms24098308

**Published:** 2023-05-05

**Authors:** Laura Marinela Ailioaie, Constantin Ailioaie, Gerhard Litscher

**Affiliations:** 1 Department of Medical Physics, Alexandru Ioan Cuza University, 11 Carol I Boulevard, 700506 Iasi, Romania; lauraailioaie@yahoo.com (L.M.A.); laserail_mail@yahoo.com (C.A.); 2 President of the International Society for Medical Laser Applications (ISLA Transcontinental), German Vice President of the German-Chinese Research Foundation (DCFG) for TCM, Honorary President of the European Federation of Acupuncture and Moxibustion Societies, 8053 Graz, Austria

**Keywords:** cancer, hepatocarcinoma, integrative nanomedicine, lasers, light, liver, multifunctional nanoplatforms, nanoparticles, photosensitizers, PDT, PIT, PTT

## Abstract

Hepatocellular carcinoma (HCC) is the most common type of primary liver cancer, with high morbidity and mortality, which seriously threatens the health and life expectancy of patients. The traditional methods of treatment by surgical ablation, radiotherapy, chemotherapy, and more recently immunotherapy have not given the expected results in HCC. New integrative combined therapies, such as photothermal, photodynamic, photoimmune therapy (PTT, PDT, PIT), and smart multifunctional platforms loaded with nanodrugs were studied in this review as viable solutions in the synergistic nanomedicine of the future. The main aim was to reveal the latest findings and open additional avenues for accelerating the adoption of innovative approaches for the multi-target management of HCC. High-tech experimental medical applications in the molecular and cellular research of photosensitizers, novel light and laser energy delivery systems and the features of photomedicine integration via PDT, PTT and PIT in immuno-oncology, from bench to bedside, were introspected. Near-infrared PIT as a treatment of HCC has been developed over the past decade based on novel targeted molecules to selectively suppress cancer cells, overcome immune blocking barriers, initiate a cascade of helpful immune responses, and generate distant autoimmune responses that inhibit metastasis and recurrences, through high-tech and intelligent real-time monitoring. The process of putting into effect new targeted molecules and the intelligent, multifunctional solutions for therapy will bring patients new hope for a longer life or even a cure, and the fulfillment of the myth of Prometheus.

## 1. Introduction

Among non-communicable diseases, cancers are a significant public health issue worldwide and the second leading cause of death in the United States, and the frequency is growing annually [1,2].

Globally, hepatocellular carcinoma (HCC) accounts for 80% of all primary liver cancers, and according to the International Agency for Research on Cancer (IARC), a specialized cancer agency of the World Health Organization (WHO), in 2018, approximately 661,000 cases of HCC occurred worldwide [3].

According to data on primary liver cancer cases and deaths from the GLOBOCAN 2020 database, an analysis that included 185 countries, it was shown that the burden of liver cancer is important but varies in different geographical areas. It was listed as the third leading cause of cancer death in 46 of the countries reviewed and ranked as the fifth leading cause of death in another 90 countries. In 2020 only, approximately 905,700 people were diagnosed with liver cancer, of which 830,200 died with this diagnosis worldwide. The incidence of new cases of liver cancer is projected to increase by 55% between 2020 and 2040, and approximately 1.3 million people could die by 2040 from this devastating disease [4,5,6].

Liver cancer is a malignancy that includes the structure of the liver and intrahepatic bile ducts and is classified as follows: hepatocellular carcinoma, cholangiocarcinoma, hepatic angiosarcoma (hemangiosarcoma), hepatoblastoma, and fibrolamellar carcinoma. HCC, known also as hepatoma, is the most common type of primary liver neoplasm. HCC is known today as a major malignant disease with high morbidity and mortality, which seriously threatens the health and life span of patients. In most cases, HCC is caused by chronic infection with the hepatitis B virus (HBV) or hepatitis C virus (HCV). However, there are also various other factors that can increase the risk of this disease, such as liver cirrhosis due to chronic alcoholism, non-alcoholic fatty liver diseases, diabetes, obesity, chronic smoking, toxic factors, genetic causes, etc. The incidence and prevalence of the disease vary between different areas of the world, reflecting economic, cultural, demographic, biological differences and political priorities in human health. Populations in Asian and African countries have the highest incidence rates of HCC, precisely because in those areas, there is an increased prevalence of hepatitis B, C, hepatitis delta virus and hepatitis E virus infections, which greatly contributes to the occurrence of chronic complications and then leads to HCC [7,8,9,10,11,12].

The clinical manifestations of HCC are initially those of chronic HBV or HCV infection, in which the classic picture includes a decreased appetite, early satiety, unexplained weight loss, chronic fatigue, fever or low-grade fever, abdominal pain, itching, skin and scleral jaundice, dark urine, increased volume of the abdomen and the presence of abdominal fluid (ascites) that returns after the discontinuation of diuretics. Physical examination may reveal a firm-to-palp liver with a sharp edge, an irregular surface, and an enlarged spleen that looks similar to an iceberg when touched by the examiner’s fingers [13,14].

The diagnosis of HCC can be suspected especially in patients known to have chronic aggressive hepatitis infection, in addition to those with liver ultrasound images suggestive of neoformations with a very rich vascularization. This unique evolutionary feature of HCC can help us to establish an early diagnosis through imaging means in a patient monitored for chronic liver disease. Practicing multiphase computed tomography (CT) or magnetic resonance imaging (MRI) as first-line methods, with the use of imaging diagnostic algorithms, are very useful in these cases. If in the case of chronic patients with hepatitis infection or cirrhosis, who are carefully monitored, the diagnosis could be established early, in most cases (which can reach 50%), the diagnosis of HCC is discovered accidentally through medical imaging [15,16].

The revised Liver Imaging Reporting and Data System (LI-RADS) for standardization of CT and MRI images in patients with suspected HCC is an excellent tool for differentiation from other abdominal tumor entities. In patients with known cirrhosis, the detection of a liver lesion US ≥ 1 cm, then investigated by CT or MRI following the detection of a solid lesion ≥ 2 cm with signal hyperenhancement in the arterial phase with late washout, most likely confirms HCC, without the liver biopsy. Although LI-RADS has proven essential for solid liver tumor recognition, it still requires many adjustments to diagnose HCC with maximum precision compared to cholangiocarcinoma (CCA), or combined HCC-CCA lesions, but especially in the monitoring of locoregional radiotherapy [17,18,19,20,21].

Today, with the expansion of nanomedicine techniques along with the diversification of nanotechnologies, mainly through the designing of many nanoparticles (NPs), new methods for an early oncological diagnosis and appropriate treatment of HCC can be provided. CT and positron emission tomography (PET) are common imaging techniques for the diagnosis of HCC. They have a sensitivity of about 80% and a specificity of over 90%. It is known that the sensitivity and resolution capacity of CT for HCC is dependent on the concentration of the contrast substance with iodine. Advances in recent years using gold-based polyethylene NPs coated with glycol and the application of spatial harmonic imaging (SHI) to X-ray scattering images have improved the detection accuracy to only a few millimeters in diameter for the cancer cells that form HCC. Through the PET technique, positron nuclides are used to mark metabolites in the investigated organism, such as glucose as an imaging agent, which shows us the metabolic changes by capturing the imaging agent by the lesion, and thus identifies the primary tumor and metastases, at a microscopic level. This reserve method has been improved by the use of liposomes (LPs) and nanoscale-modified radionuclides to increase the image signal and the chance of detecting metastases distant from the tumor site. Transcriptomics applied to the study of HCC has recognized several molecular subtypes of this tumor. PET or CT by measuring the tissue uptake of the radiopharmaceutical 18F-fluorocholine have also been able to recognize molecular imaging subtypes of HCC with relevance in the prognosis of the disease [22,23,24].

MRI has the advantage of being more specific than CT in the diagnosis of HCC, but with the conventional contrast agent based on gadolinium-diethylenetriamine pentaacetic acid (Gd-DTPA), the sensitivity and specificity of the diagnosis are still imperfect; in this sense, today, there are many attempts to assemble contrast agents based on magnetic and superparamagnetic nanoparticles [25,26,27].

Magnetic resonance elastogram/elastography (MRE) is a unique MR method for the quantitative assessment of soft tissue elasticity and structural changes. MRE is a non-invasive technique and it can accurately measure tissue stiffness and structural deviations, but it still has some disadvantages due to high costs, the need for radiologists and specialized technicians and an additional acoustic driver. Another imaging technique, MR spectroscopy (MRS), can provide comprehensive information about the pathophysiology and metabolism of tumors, but for now, its clinical applications are limited. Multiparametric MRI and PET/MRI techniques can provide more accurate information about structural and functional changes in the tumor, detect metabolites resulting from the activity of malignant cells and the interference between neoplastic cells and the tumor microenvironment (TME). These imaging techniques with nanoparticle-based MRI contrast agents facilitate diagnosis and improve individual therapies for HCC patients [28,29,30,31].

The monitoring of surgeries and especially patients for relapse control 5 years after hepatectomy can be achieved by fluorescence molecular imaging (FMI) using nanoparticle-mediated radiopharmaceutical-excited fluorescence by europium oxide NPs, which can guide the intervention and detect the occurrence of metastases ultra-small under 1 mm [32,33].

Multimodal imaging techniques supported by nanotechnology harmonize the benefits of two or more imaging methods to counterbalance the imperfections of a single technique, providing a safer procedure for the positive diagnosis of HCC [34,35].

Among the biological parameters used for the diagnosis of HCC, alphafetoprotein (AFP) is among the most frequently used biomarkers in association with imaging techniques. Screening AFP (normal blood level for adults between 0 and 40 ng/mL) with values greater than 400 ng/mL can help clarify diagnosis, prognosis, and management. Current techniques based on nano-strategies, usually electrochemiluminescence (ECL) immunoassays, using gold NPs, hybridized with other nanomaterials are very sensitive biosensors that have replaced conventional methods in AFP detection [36,37,38].

AFP-L3% (normal value = 0 to 8 ng/mL), as the glycosylated isoform of AFP, is a more sensitive biomarker than AFP for the diagnosis of HCC and of good prognosis when the value is 15% after the second liver resection [39,40,41].

Glypican-3 (GPC-3), a polysaccharide molecule released in the serum, is another biomarker for the diagnosis of HCC. Des-γ-carboxy-prothrombin (DCP), known as “protein-induced absence of vitamin K or antagonist-II” (PIVKA-II), is an abnormal prothrombin protein with a sensitivity and specificity of 71% and 84%, respectively, associated with high portal venous invasion, tumor recurrence and metastases. For the diagnosis of HBV-related HCC, DCP is an ideal marker that should be considered. Current data indicate that the biomarkers Golgi protein 73 (GP73), GPC-3 and AFP offer much better fidelity for the diagnosis of HCC when investigated together than alone or in pairs [42,43,44].

Regarding the diagnostic algorithm for HCC, there are protocol differences precisely due to the treatment methods; in Western countries, transplantation is preferred as a method of curative therapy, in contrast to Asian countries, where locoregional intervention techniques based on imaging diagnosis prevail. Even in the case of followers of imaging protocols, the diagnosis of HCC is not always only imaging. When the imaging aspects are inconclusive even when the patient is re-examined with another technique, the clinical data together with the laboratory parameters, including the liver biopsy, are evaluated by a multidisciplinary team for a correct diagnosis [13,45,46,47].

The treatment of HCC is a complex subject because ideally, it would be desirable that each case be discussed and evaluated by a multidisciplinary team consisting of a hepatologist, oncologist, surgeon and radiologist with experience in this pathology. Today, there are updated criteria for staging, treatment and prognosis, such as the 2018 Barcelona Clinic Liver Cancer (BCLC) guidelines, and the Eastern Cooperative Oncology Group (ECOG) scale. It is widely accepted that when approaching the therapy of patients with HCC, the following must be taken into account: the clinical status, liver function, tumor size and stage. Thus, for an HCC detected in the early stage, the patient can benefit from a surgical resection, ablation or liver transplant. In an intermediate stage, a local treatment can be practiced through transarterial chemoembolization (TACE). When the detection is achieved in the late stage, chemotherapeutic agents and immunotherapy are recommended. All these novel therapeutic modalities slow down the evolution in each stage of HCC and increase the patient’s 5-year survival rate. However, due to the late diagnosis in most cases, the treatment results are still unsatisfactory [48,49,50,51,52,53,54,55,56]. Patients with advanced HCC that show severe resistance to chemotherapy have been treated in the last few decades using traditional therapy with the tyrosine kinase inhibitor sorafenib, as first-line treatment since 2008. Immune checkpoint inhibitor immunotherapies have had remarkable anti-tumor effects, through programmed cell death-1 (PD-1) inhibitors (nivolumab and pembrolizumab), or programmed cell death ligand 1 (PD-L1) inhibitors (atezolizumab), and cytotoxic T-lymphocyte-associated protein 4 (CTLA-4) inhibitors (ipilimumab) [57]. Rizzo et al., recently published a judicious overview of all the challenges and future trends in HCC immunotherapy [58].

Different treatment modalities and new pharmaceutical products are constantly being explored and improved through the contribution of nanotechnologies that bring great hopes through innovative drug delivery systems (DDSs), by nanoparticles with multi-target-controlled attack, which is highly effective and ensures personalized treatment. Conventional antitumor drugs have many disadvantages, such as poor biodistribution and pharmacokinetics, low target selectivity, high resistance, and high toxicity to healthy tissues. Nanotechnology and newly designed DDSs have remarkably improved delivery to tumor sites by modifying the physical and biological characteristics of drugs and their nanocarriers, including in HCC [59]. In recent years, new methods and innovations, such as metal–organic frameworks (MOFs), have been designed to increase the efficacy of anticancer therapies, especially in drug delivery through drug loading and protection with small molecules, being able to combine chemotherapeutic drugs to deliver them effectively to the tumor tissue, including in the treatment of HCC [60].

## 2. Photodynamic, Photothermal and Photoimmune Therapy in HCC—The Basics

Conventional cancer therapies comprise surgical procedures, radiation therapy and chemotherapy, which may be efficient but have significant adverse effects. Newer, more prominent cancer-targeted action plans, such as the phototherapy methods including photodynamic therapy (PDT), photothermal therapy (PTT), and photoimmunotherapy (PIT), have sparked excitement and hope as synergistic multimodal cancer therapies, incorporating nanomedicine to overcome the biological barriers and to help treat cancer patients [61,62].

### 2.1. How Does PDT Work?

Light as a remedy dates back to prehistoric times, as ancient civilizations used to treat various disorders with light, including skin cancer. The implementation of “light” (visible, near-infrared (NIR) and ultraviolet (UV), as non-ionizing electromagnetic radiation) through the teamwork of photobiologists and clinicians in medical practice, which would include the elucidation of the cause of diseases, their prophylaxis and not only their diagnosis and treatment, but also for the preparation of pharmaceutical products, should be used more and more for the good of humanity, rather than just as an act or end in itself [63,64].

In the context of the high incidence of cancer worldwide, state-of-the-art PDT has entered as a standardized protocol and a minimally invasive procedure in the attempt to eradicate cancer. Some key moments along this long path were the accidental rediscovery at the beginning of the 20th century of the light-mediated killing effect in the presence of molecular oxygen on a unicellular protist incubated with acridine, the remarkable applications of phototherapy by Niels Finsen, for which he won the Nobel Prize in 1903, the definition of the phenomenon by Herman von Tappeiner as “photodynamic action”, and the development of photosensitizers (PSs), which started with Photofrin, as the most widely implemented PS worldwide [64,65].

PDT is clinically authorized for certain neoplasms and consists of the systemic or local administration of a non-toxic substance called a photosensitizer (PS) that should accumulate in the target tissue, which by illumination with a certain wavelength and appropriate energy in the presence of intracellular molecular oxygen will trigger PS photoexcitation and generate reactive oxygen species (ROS), initiating a cytotoxic effect. The intrinsic mechanism is that PS excited at certain wavelengths of the applied light source, in the presence of oxygen, releases more free radicals and various oxidation products with high cytotoxic potential, which will lead to cell death in irradiated cancer tissues. The wavelength of light must excite PS and it immediately reacts in the presence of oxygen with a nucleic acid, protein or unsaturated lipid from the underlying layer, and generates radicals that are not stable by proton or electron transfer, as a result of electron extraction or relocation to another molecule and/or ROS (type I reaction), for example the superoxide (•O^−^_2_), the hydroxyl radical (•OH) or hydrogen peroxide (H_2_O_2_); or, through energy transfer (type II reaction), the PS may be generated in the presence of molecular oxygen, singlet oxygen, also named dioxygen (singlet) or dioxidene O=O (^1^O_2_), which is very reactive, an important cytotoxin at high oxygen concentrations in this procedure. The ratio between type I and type II photoreactions is oxygen-dependent, involves unstable species and depends on the PS concentration, the amount of irradiation energy and it can be said that the whole process of ROS generation and tumor destruction is not yet fully understood, due to the complexity and the diversity of the degradation pathways of the most significant biomolecules upon one-electron oxidation and singlet oxygen reactions. In summary, the operating phases of PDT are as follows: (a) the administration of PS in the absence of radiation by a systemic or by a topical route; (b) when a sufficient amount of PS has already penetrated the cancer cells, the PS is photoactivated in a distinct time period, during which it reaches higher excited states, but without destroying the adjacent healthy tissue; (c) the next phase of relaxation includes generated ROS, which will kill the targeted unhealthy cells. The hit of the PDT depends on various factors, such as the deepness of light piercing into the tissue, the best assimilation of PS by the unhealthy cells, as well as the photophysical and photochemical properties of PS. Concerning the depth of light into the tissues, the absorption and scattering take place at once and are directly dependent on the wavelength applied, so PS picking up photons on the “windows of optical transparency” of those tissues will trigger deeper penetration and less attenuation during the illumination step. Conventional PS is excited usually by a higher energy (UV–Vis), but for deeper tumors, an active and safe upconverter PS that absorbs light in NIR, and releases photons in the visible and NIR ranges is required. For ideal PS, the demand for nanomaterials with powerful photoluminescence emissions and relatively long luminescence decay lifetimes (µs to ms), high sensitiveness and easy preparation is a continuous research effort [65,66,67]. Along the evolution of research and experiments on the use of light in photomedicine and the development of PDT, three generations of PSs can be distinguished, among which the last, that is, the third generation, includes compounds coupled to tumor-selective monoclonal antibodies for better pharmacokinetics and specificity with selective targeting and delayed delivery. In the latest trends, natural compounds (e.g., curcumin) along with nanotechnologies have increased clinical efficacy in tumor therapy [65].

PDT is a procedure controlled by several factors and normally involves the death of cancer cells, damage to tumor blood vessels, initiation of an antitumor immune response, and a host of inflammatory reactions in the tissue exposed to photodynamic action. Many chemicals with photosensitizing properties have been researched and there is a current trend for the study and introduction of natural products, which in relation to synthetic chemicals are accounted as “green” [14,68].

PDT can induce cancer cell death via an interplay between apoptosis, necrosis and autophagy, which can concurrently take place depending on the genetic constitution of the cells, the class or concentration of PS, its intracellular location, the PDT dose and the tissue’s partial pressure of oxygen. In recent years, several other categories of cell death have been reported as atypical pathways such as paraptosis, parthanatos, mitotic catastrophe, pyroptosis, necroptosis, and ferroptosis, in addition to immunogenic cell death (ICD) as the most promising way to eradicate tumor cells by making T-cells and the adaptive immune response operative, as well as by inducing the long-term immunological memory [69,70].

PDT can not only interrupt tumor progression, but also trigger a local acute inflammatory reaction to stimulate antitumor immunity processes by the discharge of specific subsidiary inflammatory responses and abscopal effect, as a highly satisfactory anti-cancer strategy should be able to eliminate not only the initial tumors, but also prevent metastases and recurrences [71].

In this ongoing search, researchers have undertaken and succeeded through new strategies to synthesize novel nanoparticles with good biocompatibility and biosafety as well as high PDT targeting efficiency experimentally applied to HCC, as a viable and effective future treatment options [72,73].

Registered clinical trials on PDT applied in HCC can be found at ClinicalTrials.gov (accessed on 30 March 2023), which is a database of privately and publicly funded clinical studies conducted around the world [74].

In spite of important advances in PDT applied to cancer management, there are still unequivocal impediments, such as PS collection or a photobleaching effect, restricted transferal of light doses, and inefficiency in cases of shortages in the oxygen reaching tumor tissues. On the one hand, the introspection into the complex interrelationship between hypoxia-inducible factor 1 (HIF-1) and mitochondria is very important in terms of its influence on mitochondrial structure, metabolism and respiratory function, and on the other hand, the mitochondrial activation of enzymes, the respiratory chain, the complex and decoupling proteins, all of which influence HIF-1 balance and functioning, are essential for designing and applying new high-tech, effective molecular tools and interventions in cancer therapy. HIF-1 arbitrates the accommodation to hypoxia by decreasing the mitochondrial function, and thus diminishing the oxygen cellular need. Hypoxia will degenerate mtDNA and influence the expression of many genes, including HIF-1α. Meanwhile, mitochondria will generate high metabolic stress for the HIF pathway. The complex interrelationship between HIF-1 in modulating mtDNA and mitochondrial *modus operandi* under hypoxic conditions is still poorly understood and needs to be unraveled soon [75].

### 2.2. How Does PTT Work?

Methods to improve patient outcomes and even cure cancer are important challenges for modern medicine. To reduce the side effects that accompany classical cancer therapy, i.e., radiotherapy and chemotherapy, novel PTT is confirmed as one of the most promising treatments for tumor structures in the modern era. PTT relies on ablation agents, such as PS or nanomaterials with a photothermal effect, to turn light into heat that could destroy cancer cells without harming the surrounding healthy tissues. This aspect may be true because normal cells possess greater resistance and need a longer time of exposure to heat than cancer cells. PTT consists of the application of electromagnetic waves in the NIR range for the management of diverse diseases as well as cancer. PTT is in fact an extension of PDT, for which the PS is brought into an excited state with an appropriate light bandwidth, where during de-excitation, PS releases heat, i.e., high vibrational energy that will destroy those respective cells. PTT is different from PDT because it does not need oxygen to produce effects in those living cells or organs, and could use longer wavelengths, so uses less energy and is not so harmful, but could contribute to patient recovery from cancer [76,77].

The results of recent studies prove the effectiveness of the selective destruction of tumor cells by PTT and the diversification of the means for selective killing, increasing the density of the photothermal agents (PTAs) in the tumor area, and the photothermal conversion capacity. For ideal PTT results, one of the solutions would be to increase the quantity of PTAs in the tumor area and the self-regulating photothermal conversion capability. PTA should produce more heat in the tumor and achieve low photothermal conversion in normal tissues [78,79,80].

Although noble metal nanomaterials provide particularly good photothermal effects for cancer therapy, they have some disadvantages. Nanomaterials produced on the basis of gold are not biodegradable and some structures have a relatively low photosensitivity power due to the “melting effect”. The use of light in the NIR range has several benefits because it can penetrate deeply into the tissues, where water and biomolecules absorb small amounts without causing special damage to normal tissues. When PS is exposed to continuous NIR irradiation, some gold nanomaterials would undergo structural changes and the photothermal efficiency decreases [81,82].

There are a variety of types of inorganic photothermal converters (such as previously mentioned gold nanoparticles, etc.) and organic photothermal converters, such as polydopamine (PDA) particles, that are ideal in PTT administration because have good biocompatibility and do not manifest cytotoxicity [83,84].

PTT imaging used to visualize the exact area of cancer cells and track in real-time the influx of PTT agents into tumors has recently been put into operation. Polymeric nanocomposites based on aggregation-induced emission luminogens (AIEgens) have excellent potential for applications in PTT. AIEgens are used to detect cancerous lesions and for the image guidance of neoplastic management. These AIEgens emit a bright fluorescence in the aggregated state, can contribute to the increase in the image quality, have a high toxicity, and therefore bring great benefits for photoacoustic imaging and PTT. Their multiple imaging functions can provide relevant tumor site information and direct in real-time PTT for exceptional therapeutic results with minimal adverse effects [85,86,87].

**Figure 1:**
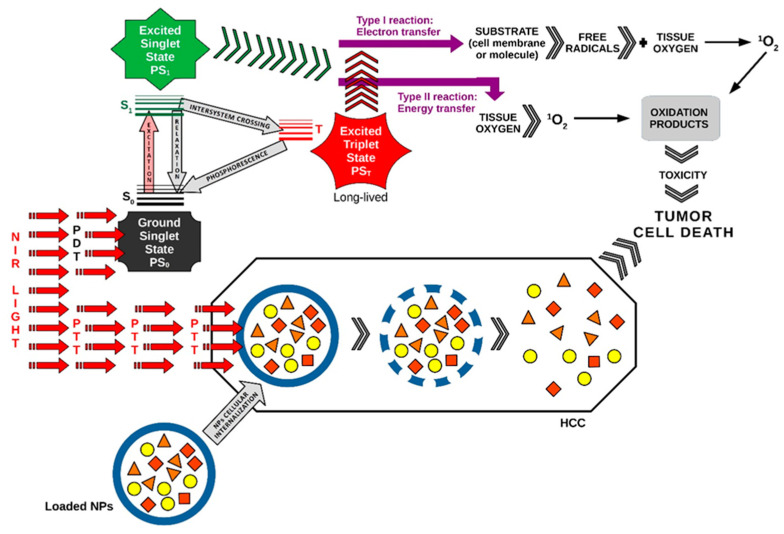
PDT and PTT applied synergistically in HCC. The pathway of action of PDT in this figure was drawn by the main author L.M.A. [65].

A picture of the main photomolecular processes in PDT and PTT applied synergistically in HCC as a combined duo-modal therapy is represented in Figure 1.

### 2.3. How Does PIT Work?

Immunotherapy is one of the most ingenious strategies for the treatment of neoplasia because it uses T-cell-activated cytokines to block the immune checkpoint blockade (ICB) or PD-1, PD-L1, and chimeric antigen receptor T cell/NK cell (CAR-T/CAR-NK). The most intensively studied are the molecules of the immune control point CTLA-4, PD-1 and PD-L1, against which inhibitory drugs have been developed. Immunotherapy for cancer includes chimeric antigen receptor (CAR)-T cells and chimeric antigen receptor (CAR) natural killer (NK) cells, i.e., CAR-NK cells, PD-1, and PD-L1 inhibitors. Through immunotherapy, the activation of the immune system is achieved to selectively control tumor growth. With all the enthusiasm and hope in these immunosuppressants, clinical trials show partial benefits, with the efficacy being limited for patients with solid tumors, and in some cases, very serious adverse reactions, such as cytokine storms, have been reported. Current concerns in the clinical therapy of solid tumors are oriented towards the discovery of new methods by which to destroy the cancer cells without affecting the patient’s immunity and with as few adverse reactions as possible [88,89,90,91,92].

Despite all the exceptional advances in the therapy of solid cancers by ICB, complete suppression of unresectable, advanced, or metastatic HCC cannot be effectively achieved due to multiple factors, including the complex immune TME. The TME is an ecosystem that includes, in addition to the respective tumor cells, various other infiltrating immune cells, fibroblasts, non-cellular structures, stromal and neovascular elements. The vessels at the level of the tumor have a different conformation compared to those of normal tissues, being strongly contorted, with various malformities, induced by rapid growth rates through the abundance of the vascular endothelial growth factor (VEGF) secreted by the tumor cells. Local oxygen deficiency, uneven distribution of nutrients and exacerbation of aerobic glycolysis with increased lactate levels lead to decreased pH in the extracellular matrix (ECM). On the other hand, HIF-1 participates in the upregulation of glycolytic enzymes, lactate transporters and excess glucose, thus amplifying acidosis. Rapid proliferation of the tumor mass leads to the compression of local vessels with increased permeability and interstitial fluid pressure (IFP). Immunologically, the TME resembles an ineffective niche, with an abundance of innate and adaptive immune cells interspersed between endothelial cells, extracellular matrix proteins, and other tumor-supporting elements, all of which prevent T cells from contacting tumor antigens and trigger appropriate immune responses. All these elements, together with acidosis, hypoxia and IFP, are specific to the TME and constitute opposing factors to the therapeutic agents that modulate the immune process [93,94,95,96,97].

PIT is a top-grade treatment method involving one or two completely different strategies; the first course of action uses PS in a stable form, already oxidized outside the body and then injected into the body. After administration, the PS concentrates specifically in the structure of the tumor tissue and changes the architecture of the tumor antigens, and the immune system will recognize the foreign tumor cells and will be prepared to eliminate them. A second strategy involves anchoring the PS to an antibody, creating a form of photoimmunoconjugate (PIC) so that the administered drug precisely hits the tumor tissue. This antibody specifically seeks out and targets a protein on the surface of the tumor, and by exposure to a non-thermal laser radiation source (e.g., 690 nm), the PS (dye) is activated, and the tumor cell membrane will be degraded, so the tumor will disintegrate with negligible alteration of the surrounding healthy tissue. PIC targets the most sensitive structures of cancer cells, namely the lysosomes, which it ruptures, thus releasing vesicles with lytic enzymes with the role of self-digestion [98,99,100].

Sustained photoactivation therapy through PDT and PTT is a modern way of ablating many forms of cancer based on PS and photothermal conversion agents. These two techniques work in tandem, in that PDT increases the responsiveness of tumor cells to PTT by altering the TME and the heat produced by PTT increases blood supply and can improve oxygen delivery, thereby increasing the efficacy of PDT. In contrast, PIT is a more refined method than traditional PDT, as it uses the ability of antibodies to target the tumor-specific antigen with high precision, increasing the immune response, cell apoptosis, autophagy or necrosis induced by phototherapy. PIT can significantly reduce tumor size by killing many cells and by enhancing the host’s immune response. PIT is much more advantageous than monotherapy because it can also assemble chemotherapeutic agents or be combined with other cancer immunotherapy methods. Through PIT, it is possible to dissipate primary tumor formations, but also eliminate or reduce systemic metastases, thus preventing recurrences [101,102].

Near-infrared photoimmunotherapy (NIR-PIT) is another recently advanced method for cancer treatment based on photochemical mechanisms. An antibody-photon absorber conjugate (APC) is used, which is stimulated by laser radiation in the NIR. The core of APC is a specific monoclonal antibody (mAb) that targets the receptor or antigen on the surface of tumor cells. Following a high concentration of APC and subsequent focal projection of NIR light, the functional structures of the targeted cell membranes will be destroyed, and a prompt and characteristic photochemical ICD will occur without affecting the neighboring receptor-negative cells. Tumor cell debris generated by PIT can function as antigens, reawakening the antitumor immune responses. Meanwhile, untrained antigen-presenting dendritic cells will mature, then initiate an anti-tumor immune response directed at the host [103,104,105].

The efficiency or performance of PIT depends to the greatest extent on the characteristics of the PS. There are many studies to discover and perfect the physicochemical properties of PSs. After administration, PS should selectively concentrate on cancer cells and, in a very small amount, on healthy tissues. We are looking for new PSs of natural origin, especially from plants. Ideal PS to be applied for diagnostic or therapeutic purposes for cancer pathology, in general, not only for PIT, must possess the following specific values: increased absorption coefficient to penetrate deeply into tissues in the visible spectrum, mainly in the red (R) region of the spectrum, or NIR (650–800 nm) range; high quantum capacity of triplet state formation; be easily available as a pure compound; to be able to achieve a high concentration in the fixed place; to have good biocompatibility, optimal pharmacokinetic properties and constant photoinduced release of singlet oxygen or other ROS; its chemical properties must be carefully determined in advance so that it is not harmful (low toxicity in the absence of light, and easily excreted from the body after treatment); and convenient costs. The absorption bands of PS should not overlap with the absorption bands of endogenous dyes such as melanin, hemoglobin, and water absorption bands in the NIR region. The largest variety of PSs used for PIT in cancer eradication includes porphyrins or their derivatives. Porphyrins have a light absorption spectrum structured in an intense and narrow absorption peak at a wavelength of about 400 nm (the Soret or B band), and other less intense Q bands at 500–650 nm. Other photosensitizers use chlorophyll derivatives with a chlorine backbone, in which the Q bands are at longer wavelengths (500–700 nm). Phthalocyanine dye IR700 (IR700) is the most common PS photo-immunoconjugate used in fluorescence imaging and PIT tumor therapy because it is synthetic and has a high absorption capacity in the NIR range [106,107,108].

Clinical studies with the NIR-PIT technique began worldwide in 2015, and the first clinical research (NCT02422979) using the target antibody cetuximab saratolacan (cetuximab conjugated to IR700, or anti-EGFR-IR700 dye conjugate, also known as RM-1929) to target the epidermal growth factor receptor (EGFR) in patients with recurrent head and neck squamous cell carcinoma (rHNSCC) was successfully concluded in 2019, demonstrating the effectiveness and safety of the method and of the product [109].

Currently, there are already ongoing phase 3 clinical trials with NIR-PIT for targeting EGFR using RM1929/ASP1929, i.e., the anti-EGFR antibody conjugate (cetuximab) plus the photo-absorber IRDye700DX (IR700). NIR-PIT has been given fast-track recognition by regulators in the USA and Japan. A diversity of imaging methods, including direct IR700 fluorescence imaging, could be applied to keep track of NIR-PIT [99].

A new clinical trial was initiated in December 2020 for the therapy of carcinoma with metastatic squamous cells that express EGFR on their surface. In this study, the antibody-dye conjugate ASP-1929 is administered in association with anti-PD1 checkpoint therapy [110].

Cancer therapy by ICB has been developed as a new technique that has revolutionized medicine by removing tumor cells that hinder the antitumor immune response. However, it has been found that only a limited number of cancer patients can benefit from this modern therapy. PDT and PTT are already known to have local beneficial effects by using PSs that can generate heat and ROS upon laser irradiation, inducing the death of immunogenic cells and opening the way for antigen-presenting cells to activate and promote a normal immune process by facilitating the infiltration of T cells into the tumor. Although ICB has emerged as a promising type of immunotherapy in advanced HCC, this ICB-only therapy is unsatisfactory with a remission rate of less than 20%. As a result, combination therapies are needed to improve therapeutic efficacy. For example, researchers have experimentally tested a targeted and ROS-sensitive micellar nanoplatform to achieve two ICD-inducing modalities by synergistic chemo-photodynamic therapy (chemo-PDT) combined with anti-PD-L1. The micellar nanoplatform was co-loaded with an aggregation-induced emission photosensitizer and paclitaxel (PTX) and was prepared for light-triggered disassembly and on-command drug release for synergistic chemo-PDT combined with anti-PD-L1, assessed both in vitro and in vivo. Chemo-PDT outstandingly increased the expression of PD-L1 on the surface of the tumor cells, which synergized with anti-PD-L1 monoclonal antibodies and set in motion an abscopal effect, generating a long-term immunological memory to hold back the tumor recurrence and metastasis. The combination of micelle-mediated chemo-PDT together with anti-PD-L1 monoclonal antibodies can synergistically enhance systemic antitumor effects and opens new avenues for novel nanomedicines with precise controlled release available for use in successful multimodal therapy for HCC [111]. NIR-PIT has become a promising therapy method for several types of cancer because it targets cancer cells, immunoregulatory cells, or both. This technique is particularly advantageous compared to the classic methods because it removes cancer cells and at the same time promotes the immune response of the host with the protection of healthy tissues in the vicinity. Photomedicine, especially through PIT, introduces the perspective of using the immunogenic action of ICD together with various other conventional treatment methods, thus offering an optimistic future for the direct destruction of primary neoplastic cells, but also the relaunch of the suppressed immune system with the aim of increasing the survival rate and even healing cancer patients. So, combining ICB therapy with PTT-PDT could increase the capacity of the antitumor immune response and stop tumor metastasis and recurrence [112,113].

## 3. Investigational Studies Using Multifunctional Platforms for PTT in HCC

Although PTT could have high feasibility for practical applications in tumor treatments, it is restricted from a practical point of view due to the short penetration distance of NIR radiation, as well as the low concentration of nanomaterials detected at the site of the tumor. Zhou et al. examined the impact of transcatheter intra-arterial (IA) infusion of lecithin-modified bismuth (Bi, chemical element with atomic number 83) nanoparticles (Bi-Ln NPs), along with interventional PTT (IPTT) on HCC. Hydrophobic Bi nanoparticles and lecithin were emulsified, resulting in Bi-Ln NPs, whose photothermal transformation and cytotoxicity were subsequently evaluated by infrared imaging and 3-(4,5-Dimethylthiazol-2-yl)-2,5-diphenyltetrazolium bromide) assays or MTT tests. Twenty-four VX2 HCC rabbits were included in the study, randomly divided into four groups, as follows: the first group or A (IA Bi-Ln NPs + NIR Laser) was treated with Bi-Ln NPs by IA infusion and NIR lasers; the second group or B (IV Bi-Ln NPs + NIR Laser) received intravenous (IV) infusion of Bi-Ln NPs and NIR lasers; the third group or C (IA PBS + NIR Laser) was treated with phosphate-buffered saline (PBS) by IA infusion and NIR lasers; and the last group or D (IA PBS) received only PBS by IA infusion, without laser treatment. The necrosis rate and viability of each tumor were rated one week after treatment. The first group A treated with IA Bi-Ln NPs + NIR lasers displayed a remarkably superior tumor inhibition rate (TIR) of 93.38 ± 19.57%, a higher tumor necrosis rate of 83.12 ± 8.02% and an increased apoptosis rate of (43.26 ± 10%) after treatment, compared to all other groups, so it could be concluded that IA transcatheter infusion in combination with IPTT is reliable and effective in killing tumor cells and stopping tumor progression, and could be considered a new and important option for the management of HCC in the near future [114].

Giammona et al., imagined and tested an intelligent composite nanosystem for NIR-triggered chemo-phototherapy in HCC [115]. They built a hybrid nanosystem capable of delivering to a well-designated region a large quantity of anticancer drugs (sorafenib or lenvatinib, which do not dissolve in water), and heat (hyperthermia) in a remotely controlled way. The scientists integrated into a special nanosystem the gold nanorods (AuNRs) for their best NIR photothermal conversion properties with a galactosylated amphiphilic graft copolymer (PHEA-g-BIB-pButMA-g-PEG-GAL) specially invented to identify liver cells that make too many copies of the asialoglycoprotein receptor (ASGPR) overexpressed on their membranes. To integrate AuNRs into the hydrophobic central part of the nanoparticles, they were covered with a layer of 12-mercaptododecanoic acid, and were loaded with drugs by the nanoprecipitation method, creating hybrid nanoparticles with a size of 200 nanometers and a loading of 9.0% *w*/*w* for sorafenib, and 5.4% *w*/*w* for lenvatinib. The versatile nanosystems released the anticancer drugs controlled and remotely, following the process of converting NIR radiation into heat. Their properties of being biocompatible in that they do not produce a toxic, harmful or immunological response in living tissues, and the synergistic repercussion of the chemo-phototherapy association, as well as receptor-mediated integration or internal co-optation, were evaluated in vitro on human HCC cell lines (HuH7 and HepG2) and compared to normal human dermal fibroblasts (NHDF). The conclusion was that these could be admirable smart hybrid nanosystems for an effective and selective dual technique as chemo-photothermal targeted therapy in HCC and other solid tumors. This advanced and original method has the great benefit of powerfully overcoming multi-drug resistance in cancer, providing a smart multi-modality system capable of discriminatively identifying and killing cancer cells through dual-modality treatment [115].

Newly developed treatment modalities, for example ferroptosis-mediated cancer treatment combined with phototherapy, promote new chances for HCC. As an iron-dependent programmed cell death, ferroptosis was distinguished from other forms of cell apoptosis by the extreme peroxidation of membrane lipids. The result of combining several therapies could be many times more effective than a single therapy modality. However, it should be considered that many therapies in progress nowadays have serious adverse effects, which could contribute to a severe decrease in the patient’s quality of life. Since combined therapy through controlled in situ tumor activation will decrease side effects and increase efficacy for precise tumor treatment, Tang et al. synthesized a GSH-activatable nanomedicine to synergistically combine the effects of PTT and ferrotherapy. The researchers applied the iron (III)-coordinated squaraine (SQ890) as a dye in the NIR range with dual functions, such as iron chelation, but also as a photothermal converter agent, enclosed with a GSH-sensitive polymer (PLGA-SS-mPEG), to obtain SQ890@Fe NPs, which are non-toxic, harmless and without any unwanted immunological response in living tissues. In the TME, SQ890@Fe-NPs displayed a photothermal effect activated by GSH, which could increase the Fenton reaction speed. The exhaustion of GSH could foster the development of ferroptosis’ effects and the multiplication of radicals will affect the configuration of the heat shock proteins (HSPs), which may increase the effects of PTT by restricting the self-protection process. The smart nanomedicine SQ890@Fe-NPs initiates a new dimension for combined tumor therapy by ferrotherapy and PTT, and thus increases the efficiency and the safeness of cancer therapy by collaboratively advancing two synergistic treatment tools, with important antitumor results proven both in vitro and in vivo [116].

Second near-infrared light-activated photothermal therapy (NIR-II PTT) is a recent tumor therapy modality that has become prominent, which applies NIR-absorbing nanomaterials in the 1000–1700 nm range and turns the photons energy into hyperthermia for tumor abscission. The positive characteristics are low toxicity, reduced photons scattering and a higher maximum permissible exposure (MPE; 1 W/cm^2^ at 1064 nm) than NIR-I light (0.33 W/cm^2^ at 808 nm) [117,118,119].

Tian et al., designed and prepared NIR-II-absorbing polyaniline nanorods (HPW@PANI Nanorods) via chemical oxidative polymerization of aniline under phosphotungstic acid, which displayed successful NIR-II absorption for hyperthermia and ablation of tumoral cells [120]. The researchers applied the HPW@PANI nanorods for in situ NIR-II PTT in the orthotopic HCC in rabbits, using optical fiber transmission of laser power. By transarterial infusion, the HPW@PANI nanorods became quickly settled into the primary orthotopic transplantation VX2 tumor in rabbits, and in situ NIR-II PTT was applied through an optical fiber inserted interventionally into the VX2 primary tumor, and 1064 nm laser energy was supplied. This protocol was tested for primary tumor ablation, remote tumor inhibition and the suppression of peritoneal metastasis. This experiment provides new hints for in situ NIR-II PTT implementation using optical fiber transmission of laser energy coupled with transarterial injection of NIR-II absorption nanomaterials to treat the deepest solid tumors [120].

As already proven, the activation of NPs in the TME brings into play synergistic curative or restorative results together with chemotherapy against many types of cancer. Huang et al. envisioned an oxaliplatin delivery system for HCC using biocompatible MIL-100 NPs. These NPs were coated with polydopamine (PDA) and NH2-PEGTK-COOH and then loaded with oxaliplatin, thereby realizing the multifunctional NP Oxa@MIL-PDA-PEGTK. The oxaliplatin-loaded metal−organic multifunctional nanoplatform is activated by light in the TME, generating cytotoxic ROS via the Fenton reaction and the delivery of the loaded oxaliplatin takes place. Simultaneously, the Oxa@MIL-PDA-PEGTK NPs can trigger hyperthermia at the tumor site under NIR irradiation. Due to more precise light-induced activation of the Oxa@MIL-PDA-PEGTK NPs, a superior (drug delivery) DD effectiveness, a more accurate targeted operation and a low off-target toxicity were noticed both in vitro and in vivo experiments. The researchers concluded that Oxa@MIL-PDA-PEGTK can serve as a new modus operandi for the management of HCC due to its superior DD efficiency and multifunctional performances, including PTT-coupled targeted chemotherapy and chemodynamic therapy [121].

A summary of the investigational studies discussed above with the potential of PTT using multifunctional nanoplatforms for the management of HCC is presented in Table 1.

## 4. Experimental Applications of PDT and PTT in HCC

Cancer in general, but HCC in particular, has become one of the main threats to human health today, despite all the efforts and advances in the field of nanomedicine. Traditional methods of surgical ablation, chemotherapy, radiation therapy, radiofrequency ablation or microwave ablation, and more recently immunotherapy, or statins in the chemoprevention of HCC are still limited by side effects, poor targeting and lack of specificity. 

Photothermal therapy was recently introduced for the treatment of cancer by using optical energy produced especially by a high-energy laser, which is absorbed by photothermal agents and generates a state of local hyperthermia, which leads to irreversible damage to tumor cells. However, most photothermal agents have effects limited by their larger size, low biocompatibility, low depth penetration, low photothermal conversion efficiency (PCE), and several undesirable effects, such as normal tissue damage, inflammation, metastasis, and sometimes tumor recurrence [122,123,124].

Li, X. et al., attempted to improve the potential of phthalocyanine (Pc) monomers as a possible PS by structural regulation with photoinduced substituent electron transfer effects, and on a metal-induced paramagnetic pathway [125]. By these techniques, they obtained three Pc compounds that exhibit excellent photothermal effects, even higher than those of indocyanine green (ICG) and methylene blue (MB). In vitro and in vivo studies have demonstrated that these unique Pc compounds are very promising for PTT in cancer phototherapy. The scientists investigated the potential effects of modified structural variants of Pc in PTT. The first experiment was conducted with copper phthalocyanine 1 (PcC1) injected into the S180 tumor in mice, after which the area was irradiated with a 685 nm laser for 10 min at a power density of 0.2 W/cm^2^. After PTT, the temperature increased to approximately 53 °C, compared to 36 °C, the temperature of tumors irradiated only with the laser. At the same time, PTT also inhibited the tumor growth volume. The phototherapeutic effect mediated by amino substituted Pc groups (PcA1 and PcA2) was studied in vitro on human hepatocarcinoma (HepG2) cells incubated with these agents and exposed to 730 nm, 1.0 W/cm^2^ laser irradiation for 10 min. Cell death occurred in 90% of cells for both PSs but was not altered by darkness or laser irradiation. At the same time, the temperature inside cells treated with PcA1 reached 45 °C (hyperthermia), compared to the temperature increase in PcA2-treated cells, which remained as low as in untreated cells. After axial replacement of Pc with piperazine (PcB1), incubation of HepG2 cells and laser irradiation, the effect of PTT was 70%, and that of PDT 30%. These results could stimulate research on the development of new innovative multifunctional phototheranostic agents, based on Pc molecular dyes [125].

Because HCC has become one of the most common malignancies in the world, and the conventional therapies of surgical resection, radiotherapy and chemotherapy did not yield the expected results, methods such as nano-PTT and biotherapy have been accepted in recent years for the treatment of HCC. In a recent study, Ma et al. attempted to combine the efficiency of nanoscale DDss with those for targeting chimeric antigen receptors (CARs)-T cells. The receptors considered chimeric are those that combine the functions of antigen binding, but also those of activating T cells in a single receiver. The authors prepared a nanomaterial by modifying CAR-T cells to recognize the highly expressed human protein Glypican-3 (GPC3) on the surface of HCC cells, by extracting their membranes and coating them with mesoporous silica loaded with IR780 fluorescent dye nanoparticles, which can produce heat and fluorescence under laser irradiation. In vitro studies have been conducted using the novel nanoparticle-coated CAR-T cell membrane upon irradiation of human HCC cell lines Huh-7 and SK-HEP-1 cultures with an 808 nm laser (0.6 W/cm^2^, for 5 min). To test the in vivo antitumor effect, the Huh-7 cells were subcutaneously injected into the flanks of 5-week-old male BALB/c-nu mice. The mice were divided randomly into five groups (each group included five mice): (1) the control (saline) and other four tested groups with different experimental protocols. The IR780 dosage was 1.5 mg/kg in groups 2, 3, 4 and 5. Saline and various nanomaterials were injected intravenously. After 24 h post-injection, the tumor sites of mice in groups 4 and 5 were irradiated by an 808 nm laser (0.6 W/cm^2^, 5 min). The administration and irradiation were conducted every 3 days for 19 days. This study demonstrated photothermal (PT) antitumor capabilities and precise targeting of malignant cells, foreshadowing an optimistic procedure for HCC therapy [126].

Glycoprotein P (P-gp) is a protein produced under the action of the gene multidrug resistance mutation 1 (MDR1) and is normally found in different types of cells, with the role of helping the transmembrane transport of toxic products; it is found in high concentrations in liver cancer cells and directly participates in the phenomenon of resistance due to some antineoplastic drugs. The P-gp, as a transmembrane efflux pump, discharges drugs from the cytoplasm to the extracellular domain, causing the intercellular drug concentration reduction and the chemotherapeutic availability suppression. P-gp is a multidrug efflux transporter that functions to protect cells from xenobiotics by exporting them out through the plasma membrane to the extracellular space. P-gp inhibitors have been developed in an attempt to overcome P-gp-mediated MDR; however, their lack of specificity and dose-limiting toxicity have restricted their clinical effectiveness [127,128,129].

In a recent study Li, S. et al. used molybdenum disulfide (MoS_2_) nanoparticles and layered MoS_2_ hollow spheres (LMHSs), which they loaded with ICG and curcumin (Cur), thus creating a nanoplatform (ICG&Cur@MoS_2_) for the delivery of photothermal and photodynamic therapy to inhibit P-gp action in HCC patients [130]. Through various experiments, the authors tested the antitumor capacity of the ICG&Cur@MoS_2_ nanoplatform and the inhibitory power of curcumin on P-gp. The PT effect of ICG&Cur@MoS_2_ was experimentally tested by irradiating with an 808 nm NIR laser at 2.0 W/cm^2^ for 5 min some samples of ICG&Cur@MoS_2_ at different concentrations, compared to pure water as a control group. The PT properties of ICG&Cur@MoS_2_ in vitro after 5 min of irradiation did not change the water temperature, while the temperature of the group with 1.00 mg/mL increased to 60.7 °C. So, the ICG&Cur@MoS_2_ NPs showed an excellent photothermal effect. For the acute in vivo toxicity study, four groups of randomly assigned female mice were used, which were injected with ICG&Cur@MoS_2_ NPs via the tail vein at different doses. Mice treated with PBS were part of the control group. After 20 days, the mice were sacrificed, and histopathological examination found no inflammatory or fibrotic changes. Blood samples for complete blood count, biological samples of liver and kidney lesions were evaluated. No changes, including appetite or weight loss, were found for either group, including the control group. These results demonstrated that the toxicity of ICG&Cur@MoS_2_ NPs was relatively low. At the same time, ICG&Cur@MoS_2_ NPs were shown to exhibit remarkable biocompatibility.

The effect of PTT-PDT in vitro was investigated by culturing human hepatocellular carcinoma HepG2 cells for 12 h with methyl-thiazolyl-tetrazolium (MTT), followed by the addition of either ICG, MoS_2_, ICG@MoS_2_, or ICG&Cur@MoS_2_ and the irradiation of cells with an 808 nm NIR laser (1.2 W/cm^2^) for 3 min. Absorbance was measured with a microplate reader at 492 nm. After Cur was removed from ICG@MoS_2_, the inhibition of HepG-2 cell proliferation was evident. Cell viability in the ICG&Cur@MoS_2_ + NIR group was significantly lower than that in the ICG@MoS_2_ + NIR group (75.3% vs. 81.2%) at ICG concentrations of 0.5, 5, 25, 50 μg/mL. The results can be attributed to the synergistic effect of PTT-PDT and of P-gp inhibition. The TUNEL (terminal transferase-mediated dUTP nick end-labeling) assay was used to explore cell death. Apoptosis was significant in the ICG@MoS_2_ + NIR group, induced by heat and ROS release.

The effect of PTT-PDT in vivo was studied in female ICR mice injected with H22 tumor cells. When the tumor grew to 200 mm^3^, the mice were injected in the tail vein and then randomly assigned to the following six groups: (I) saline; (II) ICG&Cur@MoS_2_ + NIR; (III) ICG&Cur + NIR; (IV) ICG@MoS_2_ + NIR; (V) MoS_2_ + NIR and (VI) treated only with NIR. In addition, 10 h after the injection, the NIR groups received therapy in the tumor area with an 808 nm NIR laser (1.2 W/cm^2^) for 5 min. Body weight and tumor size were monitored. In the control group, the weight of the mice increased by increasing the size of the tumor. In the ICG@MoS_2_ + NIR group, the tumors were significantly smaller than in the MoS_2_ + NIR group. PTT combined with PDT had a satisfactory effect. However, compared with the ICG@MoS_2_ + NIR group, the tumors in the ICG&Cur@MoS_2_ + NIR group were more effectively controlled. The results can be attributed to P-gp inhibition. In the research performed, Cur loaded on LMHSs was a safe and effective inhibitor of P-gp. ICG&Cur@MoS_2_ NPs can significantly improve the therapy effect.

The inhibition of P-gp by ICG&Cur@MoS_2_ was investigated by Western blotting, Q-PCR and immunofluorescence assay. Compared with the control group, P-gp in HepG-2 cells treated with ICG&Cur@MoS_2_ was significantly inhibited. mRNA was significantly decreased in the ICG&Cur@MoS_2_ group, demonstrating that ICG&Cur@MoS_2_ inhibited MDR1 gene transcription, and implicitly P-gp, which is encoded by MDR1 [130].

AIEgens are a class of unconventional materials, i.e., a class of ‘heterodox’ molecules that are non-emissive or weakly luminescent in the solution state, but efficiently emit in the aggregate state; they have excellent photostability and good biocompatibility. All these features are exploited in a number of biomedical applications for clinical investigations and therapy, including tumor instances. Synthetically obtained AIEs have high costs and biocompatibility problems. In contrast, bioproduct-inspired AIEgens (BioAIEgens) can compensate in terms of their high biocompatibility, low costs, and easy preparation [131,132].

Chai et al., investigated the cytotoxic effects of a new PS, 1-[2-hydroxyethyl]-4-[4-(1,2,2-triphenylvinyl)styryl]pyridinium bromide (TPE-Py-OH) of tetraphenylethylene derivative with AIE characteristics, designed and synthesized for PDT [133]. The authors applied this novel PS in vitro to human HepG2 cells cultured and incubated at various concentrations and irradiated with a 450 nm, 30 mW/cm^2^, 1.8 J/cm^2^, 18–45 J/cm^2^ blue laser. Cell viability was significantly reduced, varying with the concentration of incubated PS and the duration of 450 nm laser irradiation. ROS generation was dependent on TPE-Py-OH. Multi-light activated PDT was applied in vivo in a randomized study on five groups of eight mice each with HCC induced by the H22 model cell line transplanted in BALB/c mice. In group 1 (TPE-Py-OH 3 IR), 50 µg/100 µL of TPE-Py-OH was injected intratumorally, then irradiated with a 450 nm laser (100 mW/cm^2^, 10 min, 60 J/cm^2^) on days 1, 3 and 5 after injection. Group 2 (TPE-Py-OH 1 IR) was injected with the same amount of TPE-Py-OH followed by laser irradiation (450 nm, 100 mW/cm^2^, 10 min, 60 J/cm^2^) only on the first day after injection. Group 3 (TPE-Py-OH non-IR) received an injection of the same amount of TPE-Py-OH, without irradiation. Group 4 (3IR) was injected intratumorally with 100μL of fetal bovine serum (FBS) and then irradiated with a laser (450 nm, 100 mW/cm^2^, 10 min; 60 J/cm^2^) on days 1, 3 and 5 after injection. Group 5 was the negative control (NC). In the group of mice treated with TPE-Py-OH and irradiated three times, the tumor size was obviously smaller, and the survival was better than in the other groups. The authors assumed that TPE-Py-OH could be stored in lipid droplets (LDs) in a relatively stable state and retain its ROS-generating capacity for more than 7 days. TPE-Py-OH can not only target LDs and mitochondria simultaneously, but it continues to remain inside the cells for about one week, so its effect can be activated by multiple irradiations after a single injection, giving rise to multiple PDT effects activated by light. Researchers hypothesize that this innovative AIE-active PS could hold promise for the follow-up and PDT ablation of HCC with uninterrupted efficacy [133].

Li, B. et al., designed the nanoparticle P(AAm-co-AN)-AuNRs@CeO_2_-Ce6(PA/Ce6) by grafting cerium dioxide (CeO_2_)-coated gold nanorods onto the surface of the temperature-sensitive polymer P(AAm-co-AN)-CTPD, and then loading the photosensitizer Ce6 onto the surface of the nanoparticles and the polymer layer, for a new modality of antitumor treatment [134]. The in vitro antitumor effect was demonstrated on the one hand by reducing the viability of HepG2 cells treated with nanoparticles and irradiation with a 660 nm laser, through which the photosensitizer Ce6 generated singlet oxygen; and on the other hand, the gold nanorods under the action of 880 nm laser irradiation succeeded photothermal conversion and induced local heating, which led to the phase transition of the polymer layer and achieved a well-controlled release mechanism. The antitumor efficacy in vivo was investigated on mice with experimentally induced tumors with HepG2 cells, randomized into six groups after injection and laser irradiation, as follows: saline solution; saline + laser (808 nm + 660 nm); PA/Ce6; PA/Ce6 + 808 nm laser; PA/Ce6 + 660 nm laser and PA/Ce6 + laser (808 nm + 660 nm). Mice weight and tumor volume were then monitored every other day, and after 15 days of treatment, the mice were euthanized. In the control group with saline solution, the tumor volume did not change and instead, the optimum inhibitory effect was observed in the group of mice irradiated with NIR. The nanoparticles had good biocompatibility, without acute toxicity. The authors concluded that under 808 nm laser irradiation for 600 s, the PA/Ce6 solution was heated to about 60 °C, which was sufficient to eliminate both cancer cells and tumor tissues. Compared with free Ce6, the ROS-mediated fluorescence of PA/Ce6 nanoparticles increased considerably. Simultaneous irradiation with red and NIR lasers destroyed the viability and motility of HepG2 cells, producing a strong antitumor effect in vitro. It was proved that PA/Ce6 could successfully decompose hydrogen peroxide under laser irradiation and effectively attenuate the anaerobic TME, opening up favorable future perspectives and avenues for the management of HCC by synergistic PTT and PDT [134].

Early diagnosis and salutary treatment for most HCC patients would offer the chance of a better prognosis. Today, the imaging methods offered by ultrasound, CT and MRI show low sensitivity or inadequate penetration depth for the diagnosis of millimeter-sized HCC. On the other hand, optical imaging through its high sensitivity, biological safety and the ability of electromagnetic scanning at the molecular level offers the chance of a precise diagnosis of HCC in the early stage. Near-infrared (NIR) photoacoustic imaging (PAI) and fluorescence imaging (FLI) have been recently manufactured for early tumor detection and recognition. PAI detects the tissue image at a depth of 5–7 cm in a localized area and provides more precise information than traditional optical imaging methods. PAI is based on the recognition of ultrasound resulting from thermal agent-induced vibration and tissue expansion after capturing specific projected light with a pulsed laser. Since PAI provides a particularly accurate resolution in deep tissue, and FLI has imaging sensitivity, the simultaneous application of these two modalities can accurately detect tissue contrasts, hemoglobin concentration, oxygen saturation, lipids and a wide variety of molecules and nanoparticles. Combined PAI/FLI offers the excellent possibility of simultaneous administration with high efficiency of PTT and PDT in tumor ablation and hypoxia relief. However, unfortunately, we still do not have a wide range of nano-agents to use imaging techniques to establish a precise diagnosis and administer targeted phototherapy. Another prominent imaging technique with high sensitivity and no depth penetration restrictions is magnetic particle imaging (MPI), which, combined with FLI, could refine the accuracy of HCC diagnosis at a very early stage. The latest studies express the potential of combinatorial biomaterials and imaging techniques for an early diagnosis of HCC [135,136,137,138,139,140,141,142].

In recent research, Qi et al. synthesized a versatile indocyanine green (ICG)/platinum (Pt)-doped PDA melanin-mimic nanoparticle designed to target C-X-C chemokine receptor type 4 (CXCR4) (called ICG/Pt@PDA-CXCR4, or IPP-c) with a special HCC-specific resolution. CXCR4, a protein that is a CXC chemokine receptor, a molecule with strong chemotactic activity for lymphocytes, the most widely expressed and involved in numerous physiological and pathological conditions, was confirmed to be highly expressed in the cell membrane of human HCC cells (HepG2 and LM3 cells). IPP-c has been very useful for PAI/FLI in clarifying the diagnosis and optical imaging-directed PTT/PDT to small orthotopic hepatocellular carcinoma (SHCC). The study confirms that IPP-c had good stability, biocompatibility and high quality of specific targeting and killing of CXCR4-overexpressing HCC cells. At the same time, SHCCs smaller than 1.2 mm in size were accurately detected by the photoacoustic/fluorescence dual-modal imaging technique both in vivo and ex vivo. Real-time guidance of PTT/PDT methods by optical imaging with the proper release of singlet oxygen after irradiation with an 808 laser and high PCE, together with minimal invasion of orthotopic SHCCs without the destruction of neighboring liver tissues or other major organs, have been successfully performed [143].

Because most patients with advanced HCC receiving chemotherapy have primary or secondary drug resistance, there is an urgent need to find new strategies to enhance the efficacy of chemotherapy. Complex treatment combining chemotherapy with other procedures could advance responses to anticancer treatment and could provide important benefits to overcome chemoresistance in these patients. In this regard, Wang et al., assembled a multifunctional nanoplatform (MnO_2_-SOR-Ce6@PDA-PEG-FA, MSCPF) using MnO_2_ as a photothermal agent and Ce6 as a photosensitizer. Sorafenib (SOR), the multitarget agent used as the first-line treatment in advanced stage HCC, was loaded and irradiated the tumor with a NIR laser applied as PTT-PDT [144]. The photothermal effect of MnO_2_ and PDA was investigated by irradiating samples of H_2_O, MnO_2_ and MnO_2_@PDA with an 808 nm laser for 10 min at a power of 1.5 W/cm^2^. The sample with MnO_2_@PDA recorded a temperature increase from 28.9 to 53.1 °C in 10 min. The PCE, i.e., the heating–cooling curve of the MSCPF platform, at an irradiation with 808 nm and a power density of 1.5 W/cm^2^ for 6 min was 52.64%. PCE together with the excellent photothermal effect of MSCPF guarantees the beneficial potential of PTT. PTT/PDT efficiency in vitro was studied on cultures of SMMC-7721 cells irradiated with a laser at 808 nm (for PTT, 1.5 W/cm^2^) or 660 nm (for PDT, 500 mW/cm^2^) for 10 min, after incubation with free Ce6, MnO_2_-Ce6@PDA (MCP) and MnO_2_-Ce6@PDA-PEG-FA (MCPF). MCPF demonstrated high phototoxicity on SMMC-7721 cells and had superior antitumor effects by combining PTT with PDT. The amount of ROS was 3.90 times higher in cells treated with MCPF than those with free Ce6, confirming that the nanoplatform fulfills its PDT task very well inside the cells. The use of the MSCPF nanoplatform loaded with SOR in combined administration with PDT/PTT and chemotherapy had a higher cell apoptosis rate (10.6%) than SOR alone (5.34%). MSCPF used in combined chemo/PTT/PDT therapy could decrease the motility and invasion of cancer cells. Investigating the antitumor action of MSCPF NPs by inducing ferroptosis and inhibiting P-gp expression demonstrated that tumor cell microstructures showed morphological changes typical of ferroptosis (smaller mitochondria, loss of mitochondrial crests, and increased membrane density). The level of SOR inside SMMC-7721 cells increased significantly, and P-gp expression decreased after MSCPF treatment, an aspect demonstrated by Western blot measurements. So, the MSCPF nanoplatform had a major role in inhibiting P-gp expression, thus leading to increased intracellular accumulation of SOR and enhanced antitumor efficiency. The anti-tumor action of MSCPF NPs was evaluated in vivo on mice bearing SMMC-7721 tumors randomly arranged in four groups, as follows: PBS (control); SOR; MCPF + irradiation at 660 and 808 nm; and (IV) MSCPF + irradiation at 660 and 808 nm (SOR concentration = 630 μg/kg). A reduction in tumor volume was observed after administration of SOR, MCPF and MSCPF. The TUNEL technique highlights the reduction in cancer cell proliferation and the increase in apoptosis. The authors claim that MSCPF, through its controlled release action and inhibition of P-gp expression, could favor the increase in the concentration and distribution of SOR inside the tumor. In addition, the increase in local heat, the level of ROS after laser irradiation with two wavelengths, and the splitting of H_2_O_2_ with the release of O_2_ would reduce tumoral hypoxia and enhance the effect of Ce6-mediated PDT and PDA-mediated PTT in the destruction of HCC [144].

Recent studies on the single effects of PTT or PDT demonstrate that they are less beneficial than coadministration using a dual action photosensitizer. Xu et al. investigated in vitro the anticancer action on HepG2 cells of the supramolecular material Purp@COP administered simultaneously for both PDT and PTT. Purp@COP has photodynamic and photothermal effects because it includes covalent organic polymers (COPs) that have a highly cross-linked porous structure, multiple functions similar to the metal–organic framework, it is insoluble in organic solvents, and the Purp object that is methyl viologen (1,1′-dimethyl-4,4-bipyridinium dichloride (6Cl,7Cl)), anchored to COP by a physical load. During the preparation of the Purp@COP material, platinum nanoclusters (Pt^2+^) were incorporated, creating a phthalocyanine quasi-backbone with a platinum coordination center, an excellent structure with light absorption properties that ensured the photodynamic effect with excessive ROS production and photothermal properties for destroying cancer cells. The authors cultured a HepG2 cell line for HCC with Purp@COP for 24 h, after which they irradiated it with an 808 nm NIR laser at a dose of 1 W/cm^2^ for 10 min. The results demonstrated in vitro that cell resistance and colony formation capacity were reduced, and the percentage of apoptotic cells increased under the influence of PTT/PDT effects in the presence of Purp@COP supramolecular material [145].

A synthesis of the experimental studies presented above regarding the combined effects of PTT and PDT in HCC is shown in Table 2.

State-of-the-art experimental applications of PDT, PTT and PIT for synergistic therapy in HCC, one of the most lethal forms of cancer, were presented, highlighting the special role of future combination therapies in clinical applications for personalized interventional treatments.

Extensive randomized clinical trials should utilize these novel nanoplatforms, nanotechnologies, and ingenious DDSs summarized above to enhance the extraordinary regenerative capacity of the liver, which, as in the myth of Prometheus, was recognized by the ancient Greeks as extraordinary and inexhaustible.

The nanomedicine of the third millennium should be set in motion and modulate with the help of light, the immunological and abscopal interventions that block the phenomena of tumor recurrence and metastases in HCC, so that the patient leaves the hospital cancer-free.

## 5. Conclusions

With all the important advances in 21st century nanomedicine, researchers are still struggling to face all the major barriers to HCC management in terms of early diagnosis, tumor growth arrest and ablation, prevention of metastases, and prolongation of patient survival.

Advancing multifunctional systems by combining and tailoring FDA-approved drugs for HCC to overcome MDR to chemotherapy and monomodal therapies with the latest combined procedures such as PDT, PTT and PIT is an important goal.

Innovative theranostic systems that provide high-quality P-gp blockade and near-infrared fluorescence imaging-guided chemo-photothermal therapy provide new perspectives on the development of novel multi-operational platforms and smart DDDs loaded with anticancer drugs with very good photothermal conversion for synergistic action of PDT, PTT and PIT, but also high-tech imaging and immunomodulatory properties in HCC. 

The characteristics of the new engineered NPs have been intensively studied in recent years from all points of view, including TME, FL imaging in vitro, photothermal effects in vitro and in vivo and their controlled release based on recently discovered molecular processes in the pathophysiology of HCC.

The cutting-edge scientific experimental studies in HCC highlighted in this review on combined multimodal therapies such as PDT, PTT, and PIT, as well as the molecular and cellular mechanisms revealed, may improve the current impact of anticancer management and open the prospect of successful applications. The process of implementing new targeted molecules and smart, multifunctional solutions for therapy will bring patients new hope for a longer life or even a cure, and the fulfillment of the myth of Prometheus.

## Figures and Tables

**Table 1:** PTT combined with hybrid nanosystems as multimodal therapy in HCC.

NPs	Experimental Protocol	Conclusions	References
Lecithin-modified Bi nanoparticles **(Bi-Ln NPs)**	24 VX2 HCC rabbits were randomly divided into 4 groups, as follows: group A (IA Bi-Ln NPs + NIR Laser); group B (IV Bi-Ln NPs + NIR Laser); group C (IA PBS + NIR Laser); and group D (IA PBS).	Group A displayed a remarkably superior TIR, a higher tumor necrosis rate and an increased apoptosis rate, compared to all other groups. Transcatheter IA combined with IPTT is safe and effective in killing tumor cells and inhibiting tumor growth, and could be applied in HCC soon.	[114] (Zhou, J. et al., 2020)
Galactosylated polymer/gold nanorod nanocomposites: PHEA-g-BIB-pButMA)-g-PEG-GAL embedding AuNRs-C12 and loaded with SOR or LEN i.e., **(SOR-NPs) and (LEN-NPs)**	Anticancer activity of the SOR-NPs and LEN-NPs was assessed for two HCC cell lines and compared to NHDF. Cells were seeded in a 96-well plate with a density of 1.0 × 104 cells per well (200 μL) and grown for 24 h in a DMEM. Successively, the medium was replaced with a dispersion of SOR-NPs or LEN-NPs. Drug release profiles were evaluated by irradiating the dispersion of nanoparticles with a diode NIR laser (λ = 810 nm, at a power = 0.7 W/mL for 5 or 20 min, at different scheduled time intervals (0 h, 1 h, 3 h, and 6 h). Anticancer activity of the SOR-NPs and LEN-NPs was studied with (810 nm diode laser for 300 s (for SOR-NPs P = 10 W, for LEN-NPs P = 6 W) and without NIR exposure.	The hybrid nanosystems SOR-NPs and LEN-NPs (diameter of about 214 nm and 148 nm, respectively), demonstrated optimum NIR photothermal conversion, high drug loading, and excellent NIR-driven drug release enhancement. These smart drug-loaded hybrid nanosystems for NIR-triggered chemo-phototherapy in HCC with high biocompatibility entered the cancer cells overexpressing ASGPR, where they can release heat and drugs. The innovative approach potentially overcomes MDR in cancer and is a multimodal tool capable of selectively recognizing and killing HCC cells through dual-mode therapy.	[115] (Giammona, G. et al., 2022)
SQ890 was encapsulated with a GSH-sensitive polymer (PLGA-SS-mPEG) to obtain biocompatible **SQ890@Fe NPs**	Treatment of HepG-2 cells with different concentrations of SQ890@Fe NPs showed the potential to infiltrate cancer cells. The tumoricidal efficacy of SQ890@Fe NPs in vivo in mice, after anesthetization and 808 nm illumination (1 W/cm^2^) for 5 min, increased the temperature at the tumor sites above 50 °C in 5 min, which led to tumor ablation.	Combined PTT and ferrotherapy on SQ890@Fe NPs demonstrated increased efficacy and safety in the treatment of HCC both in vitro and in vivo by mutually promoting two treatment mechanisms.	[116] (Tang, L. et al., 2022)
**HPW@PANI nanorods**	HPW@PANI nanorods were applied for in situ NIR-II PTT in orthotopic HCC in rabbits. 1064 nm laser energy was delivered through an optical fiber inserted interventionally into the VX2 primary tumor, with the protocol being tested for different laser power densities for primary tumor ablation, inhibition of distant tumors, and suppression of peritoneal metastases.	HPW@PANI nanorods prepared via oxidative chemical polymerization exhibited strong NIR-II absorption, higher photothermal conversion efficiency, and excellent biocompatibility. In vivo experiments proved that in situ NIR-II PTT could ablate primary tumors, inhibit distant tumors, and suppress peritoneal metastasis, opening new avenues for the management of deeply camouflaged solid tumors.	[120] (Tian, C. et al., 2022)
Multifunctional **Oxa@MIL-PDA-PEGTK NPs**	After preparation, the Oxa@MIL-PDA-PEGTK NPs were tested for the cumulative release of oxaliplatin and the photothermal effect at different concentrations of NPs using CW irradiation with an 808 nm laser (for 400 s, at a power density of 1 W/cm^2^). HCC cell line MHCC97H, HCC cell line PLC/PRF/5 and the normal hepatic epithelial cell line L02 were used. Cancer cells were incubated with 100 μg/mL Oxa@MIL-PDA-PEGTK NPs for 4 h, and either left untreated or exposed to 808 nm laser irradiation for 10 min at a power density of 2.0 W/cm^2^. PLC/PRF/5 cells (1 × 10^6^) were diluted in 100 μL PBS and injected subcutaneously into the right axillary region of male BALB/C-nu mice (5 weeks old). Biodistribution of NPs and photothermal imaging, in vivo tumor growth inhibition, and biochemical examination and pathological analysis of mice were studied.	This multifunctional NP-based DDS could effectively deliver chemotherapeutic agents to tumors. In vitro experiments demonstrated Fe^2+^ release at tumor sites, increased ROS generation in laser-irradiated cancer cells, while exhibiting low cytotoxicity in non-cancerous cells, facts confirmed by in vivo studies. Design of novel TME-responsive nanoplatforms will soon improve the cure of HCC.	[121] (Huang, R. et al., 2023)

**Table 2:** Experimental applications of PTT and PDT for synergistic therapy in HCC.

Photosensitizer	PTT and PDT Protocol	Conclusions	References
Structural variants of phthalocyanine (**Pc**)	In vivo PTT efficacy of Pcs on S180 tumors was tested. Pcs were injected into the S180 tumor in mice, after which the area was irradiated with a 685 nm laser for 10 min at a power density of 0.2 W/cm^2^.	By structural adjustment, the authors obtained three Pc derivatives with PTT activity against human HCC. PcC1 had a very good in vivo PTT effect against S180 tumors in carrier mice. Pc molecular dyes are suitable for PTT, for the advanced promotion of Pc molecular dye-based multifunctional phototheranostic agents.	[125] (Li, X. et al., 2018)
CAR-T cell membrane-coated nanoparticles (**CIMs**)	In vitro antitumor effect was investigated on Huh-7 cell cultures incubated with CIMs, and in vivo after abdominal tumor development with the same cells in BALB/c-nu mice irradiated with an 808 nm laser (0.6 W/cm^2^, for 5 min).	CIMs demonstrated very good targeting and PTT efficacy, giving rise to a promising method for the treatment of HCC.	[126] (Ma, W. et al., 2020)
**Nanoplatform ICG&Cur@MoS_2_**	The PTT properties of ICG&Cur@MoS_2_ NPs were tested in vitro by irradiation with an 808 nm laser at 2.0 W/cm^2^ for 5 min on ICG&Cur@MoS_2_ samples at different concentrations. The effect of PTT-PDT in vitro was investigated after irradiation of different groups of HepG-2 cells incubated with ICG, MoS_2_, ICG@MoS_2_ or ICG&Cur@MoS_2_. The effect of PTT-PDT in vivo was investigated by a randomized study on six groups of mice with abdominal tumors induced by experimental H22 cell line. The NIR groups were irradiated in the tumor area with an 808 nm laser (1.2 W/cm^2^) for 5 min, at 10 h post-injection. Both body weight and tumor size were measured and recorded carefully.	Cell viability in the ICG&Cur@MoS_2_ + NIR group was significantly lower than that in the ICG@MoS_2_ + NIR group. The results can be attributed to the synergistic effect of PTT-PDT and P-gp inhibition. ICG@MoS_2_ + NIR group tumors were significantly smaller than in MoS_2_ + NIR group. Compared with the control group, P-gp in HepG-2 cells treated with ICG&Cur@MoS_2_ was significantly inhibited.	[130] (Li, S. et al., 2021)
1-[2-Hydroxyethyl]-4-[4-(1,2,2-triphenylvinyl) styryl]pyridinium bromide (**TPE-Py-OH**)	For long-term PDT in vitro, HepG2 cells were incubated with various concentrations of TPE-Py-OH, and then exposed to a blue laser with different durations (450 nm, 30 mW/cm^2^, 18–45 J/cm^2^). In vivo multiple light-activated PDT was investigated in a randomized study on 5 groups of 8 animals per group with HCC induced by cell line H22. TPE-Py-OH was intratumorally injected, and further irradiated with a blue laser (450 nm, 100 mW/cm^2^, 10 min, 60 J/cm^2^).	TPE-Py-OH as an innovative AIE-active PS could be promising for tracking and PDT ablation of HCC with uninterrupted efficacy.	[133] (Chai, C. et al., 2022)
P(AAm-co-AN)-AuNRs@CeO_2_-Ce6 **(PA/Ce6)**	The antitumor effects of PDT/PTT in vitro were studied on HepG2 cells incubated with PA/Ce6, followed by 660 nm and 880 nm laser irradiation for 600 s. The antitumor efficacy of PDT/PTT in vivo was investigated by monitoring the weight and volume of abdominal HCC in 6 groups of mice randomized as follows: IV saline solution; saline + light (808 nm + laser 660 nm); PA/Ce6; PA/Ce6 + 808 nm laser; PA/Ce6 + 660 nm laser; and PA/ce6 + light (808 nm + 660 nm lasers).	Viability of HepG2 cells incubated with PA/Ce6 was reduced after 660 nm and 880 nm laser irradiation. PA/Ce6 could decompose hydrogen peroxide under laser irradiation and attenuate the anaerobic TME, opening up favorable future opportunities for the management of HCC by synergistic PTT and PDT.	[134] (Li, B. et al., 2022)
ICG/Pt@PDA-CXCR4 **(IPP-c)**	After HepG2 cells were incubated with IPP-c NPs, they were irradiated with an 808 nm laser for 10 min compared with a non-irradiated set. BALB/c nude mice bearing orthotopic SHCC tumors received I.V. IPP-c NPs, after which they were irradiated in the area of liver tumors for 12 min with an 808 nm laser at a dose of 0.8 W/cm^2^.	CXCR4-targeted multifunctional nanoparticles for mini-invasive phototherapy of orthotopic SHCCs by real-time quantitative optical imaging guidance provide another perspective for constructing a nanoplatform for early-stage HCC phototheranostics.	[143] (Qi, S. et al., 2022)
MnO_2_-SOR-Ce6@PDA- PEG-FA **(MSCPF)**	Investigation of the photothermal effect of MnO_2_ and PDA by irradiating H_2_O, MnO_2_ and MnO_2_@PDA samples irradiated with an 808 nm laser for 10 min at a power of 2 W/cm^2^. A series of MSCPF solutions (50, 100, 200 μg/mL) at different power densities (0.5–2.0 W/cm^2^) were applied for the test, and the temperature rise was recorded by an NIR thermal camera. In vitro PTT/PDT efficiency was studied on SMMC-7721 cell cultures irradiated with a laser at 808 nm (for PTT, 1.5 W/cm^2^) or 660 nm (for PDT, 500 mW/cm^2^) for 10 min, after incubation with Ce6, MCP and MCPF. The antitumor effect of MSCPF NPs was evaluated in vivo on SMMC-7721 tumor-bearing mice randomly divided into four groups.	In comparison with H_2_O, MnO_2_ showed a significant temperature rise from 28.9 to 42.7 °C, while MnO_2_@PDA showed higher photothermal conversion efficiency, with the temperature rising from 28.9 to 53.1 °C in 10 min. MCPF demonstrated high phototoxicity on SMMC-7721 cells and had superior antitumor effects by combining PTT with PDT. A reduction in tumor volume was observed after administration of sorafenib, MCPF and MSCPF. The synergistic tumor-targeted and hypoxia-alleviated nanoplatform (MSCPF) that co-deliver sorafenib, Ce6, and MnO_2_ for combined chemo/PDT/PTT therapy proved an enhanced antitumor effect in HCC.	[144] (Wang, C. et al., 2022)
Supramolecular material **Purp@COP**	In vitro HCC cell line HepG2 was cultured for 24 h with Purp@COP, after which it was irradiated with an 808 nm NIR laser at a dose of 1 W/cm^2^ for 10 min.	The supramolecular material Purp@COP had double effects, both photodynamic and photothermal, with the suppression of the proliferation of cancer cells and their significant destruction. Purp@COP could be a PS with potential in the treatment of HCC.	[145] (Xu, W. et al., 2022)

## Data Availability

The references used to support the findings of this study are available from the first (L.M.A.) author upon request.

## Abbreviations

Aggregation-induced emission-based luminogens	AIEgens
Alpha (Greek letter, lowercase alpha)	α
Alpha-fetoprotein	AFP
Antibody-photon absorber conjugate	APC
Asialoglycoprotein receptor	ASGPR
ATP-binding cassette	ABC
Barcelona Clinic Liver Cancer	BCLC
Bismuth, chemical element with atomic number 83	Bi
CAR-T cell membrane-coated nanoparticles	CIMs
Cerium dioxide	CeO_2_
Chemo-photodynamic therapy	chemo-PDT
Chimeric antigen receptor	CAR
Chimeric antigen receptors or chimeric T cell receptors	CAR-T
Chimeric antigen receptor (CAR) natural killer cell	CAR-NK
Chimeric antigen receptor T cell/NK cell	CAR-T/CAR-NK
Chlorin e6	Ce6
X-ray computed tomography	CT
Continuous wave	CW
Copper phthalocyanine 1	PcC1
Copper phthalocyanine 2	PcC2
Covalent organic polymers	COPs
Curcumin	Cur
Cyclic adenosine monophosphate	cAMP
Cytochrome c oxidase	CCO
Cytotoxic T lymphocyte-associated antigen-4	CTLA-4
C-X-C chemokine receptor type 4	CXCR4
Decrease	↓
Des-γ-carboxy-prothrombin	DCP
Diffuse correlation spectroscopy	DCS
Drug delivery	DD
Drug delivery systems	DDSs
Dulbecco’s Modified Eagle’s medium (EuroClone)	DMEM
Eastern Cooperative Oncology Group	ECOG
Electrochemiluminescence	ECL
Epidermal growth factor receptor	EGFR
Extracellular matrix	ECM
Fetal bovine serum	FBS
Fluorescence imaging	FLI
Fluorescence molecular imaging	FMI
Functional near-infrared spectroscopy	fNIRS
Gadolinium-diethylenetriamine pentaacetic acid	Gd-DTPA
Galactosylated amphiphilic graft copolymer	PHEA-g-BIB-pButMA-g-PEG-GAL
Glycoprotein P	P-gp
Glypican-3	GPC3
Gold nanoparticles	AuNPs
Gold nanorods	AuNRs
Golgi protein 73	GP73
Heat shock proteins	HSPs
Hepatitis B virus	HBV
Hepatitis C virus	HCV
Hepatocellular carcinoma	HCC
Human dermal fibroblasts	NHDF
Human hepatocarcinoma cells	HepG2
Hydrogen peroxide	H_2_O_2_
Hydroxyl radical	•OH
Hypoxia-inducible factor 1	HIF-1
ICG/Pt@PDA-CXCR4	IPP-c
Immune checkpoint blockade	ICB
Immunogenic cell death	ICD
Increase	↑
Indocyanine green	ICG
Insulin-like growth factors-1	IGF-1
Interferon-γ	IFN-γ
Interleukin 1-beta	IL-1β
Interleukin-10	IL-10
Interleukin-6	IL-6
International Agency for Research on Cancer	IARC
Interstitial fluid pressure	IFP
Interventional PTT	IPTT
Intra-arterial	IA
Intravenous	IV
Iron (III)-coordinated squaraine dye	SQ890
IR780-loaded MSNs	IMs
Layered MoS_2_ hollow spheres	LMHSs
Lenvatinib	LEN
Light amplification by stimulated emission of radiation	LASER
Light-emitting diode	LED
Lipid droplets	LDs
Liposomes	LPs
Liver Imaging Reporting and Data System	LI-RADS
Localized surface plasmon resonance	LSPR
Magnetic particle imaging	MPI
Magnetic resonance	MR
Magnetic resonance elastogram/elastography	MRE
Magnetic resonance imaging	MRI
Maximum permissible exposure	MPE
Manganese dioxide	MnO_2_
Mesoporous silica nanoparticles	MSNs
Methylene blue	MB
3-(4,5-Dimethylthiazol-2-yl)-2,5-diphenyltetrazolium bromide) assay	MTT
Mitochondria/mitochondrial	mt
Mitochondrial membrane potential	MMP
MnO_2_-SOR	MS
MnO_2_-SOR-Ce6	MSC
MnO_2_-Ce6@PDA	MCP
MnO_2_-Ce6@PDA-PEG-FA	MCPF
MnO_2_-SOR-Ce6@PDA	MSCP
MnO_2_-SOR-Ce6@PDA- PEG-FA	MSCPF
Molybdenum disulfide	MoS_2_
Monoclonal antibody	mAb
MR spectroscopy	MRS
Multidrug resistance	MDR
Multidrug resistance mutation 1	MDR1
Nanoemulsion	NE
Nanoparticle	NP
Nanoparticles	NPs
Natural killer	NK
Near-infrared	NIR
Near-infrared photoimmunotherapy	NIR-PIT
Negative control	NC
Oxaliplatin	Oxa
Paclitaxel	PTX
P-glycoprotein	P-gp
Phosphate-buffered saline	PBS
Photoacoustic imaging	PAI
Photodynamic inactivation	PDI
Photodynamic therapy	PDT
Photoimmunoconjugate	PIC
Photoimmunotherapy	PIT
Photosensitizer	PS
Photosensitizers	PSs
Photothermal	PT
Photothermal agents	PTAs
Photothermal conversion	PTC
Photothermal conversion efficiency	PCE
Photothermal therapy	PTT
Phthalocyanine	Pc
Phthalocyanine amino-substituted A1	PcA1
Phthalocyanine amino-substituted A2	PcA2
Piperazine	PcB1
Poly(ethyleneglycol)-folate	PEG-FA
Polydopamine	PDA
Positron emission tomography	PET
Programmed cell death 1	PD-1
Programmed cell death ligand 1	PD-L1
Protein-induced absence of vitamin K or antagonist-II	PIVKA-II
Radiopharmaceutical-excited fluorescence	REF
Reactive oxygen species	ROS
Recurrent head and neck squamous cell carcinoma	rHNSCC
Red	R
Second near-infrared light-activated photothermal therapy	NIR-II PTT
Silver nanoparticles	AgNPs
Singlet oxygen, dioxygen (singlet) or dioxidene	^1^O_2_
Small orthotopic hepatocellular carcinoma	SHCC
Sorafenib	SOR
Spatial harmonic imaging	SHI
Superoxide	•O^−^_2_
Terminal transferase-mediated dUTP nick end-labeling	TUNEL
Theta (Greek letter, lowercase theta)	θ
Transarterial chemoembolization	TACE
Tumor inhibition rate	TIR
Tumor microenvironment	TME
Tumor necrosis factor alpha	TNF-α
1-[2-hydroxyethyl]-4-[4-(1,2,2-triphenylvinyl)styryl]pyridinium bromide	TPE-Py-OH
U.S. Food and Drug Administration	FDA
Ultrasound scan (ultrasonography)	US
Ultraviolet	UV
Ultraviolet-visible	UV-Vis
Ultraviolet A	UVA
Ultraviolet B	UVB
Vascular endothelial growth factor	VEGF
World Health Organization	WHO
